# Effect of mode of delivery on perceived risks of maternal health outcomes among expectant parents: a cohort study in Beijing, China

**DOI:** 10.1186/1471-2393-14-12

**Published:** 2014-01-13

**Authors:** Wen-Ying Li, Tippawan Liabsuetrakul, Babill Stray-Pedersen

**Affiliations:** 1 Department of Obstetrics and Gynecology, First Hospital of Tsinghua University, Tsinghua University, No. 6 Jiuxianqiao 1st Street, Beijing 100016, Chaoyang District, China; 2 Epidemiology Unit, Faculty of Medicine, Prince of Songkla University, Hat Yai, Songkhla, Thailand; 3 Division of Women and Children, Rikshospitalet, Oslo University Hospital, University of Oslo, Oslo, Norway

**Keywords:** Birth experience, Cesarean delivery, Couple, Perception, Vaginal delivery

## Abstract

**Background:**

Misperceptions regarding maternal health outcomes after vaginal delivery (VD) and cesarean delivery (CD) may contribute to the increasing trend towards CD. The effects of mode of delivery on parents’ perceived risks of health outcomes are unclear. This study aimed to compare the perceived risks of maternal health outcomes among pregnant women and their partners before and after delivery, and to evaluate factors related to inaccurate perceptions among women after delivery.

**Methods:**

Consecutive eligible nulliparous women at 36-40 weeks gestation were approached during antenatal registration for electronic fetal monitoring, regardless of whether CD or VD was planned. Eligible women were aged 18-45 years, received antenatal care and planned delivery at the First Hospital of Tsinghua University, Beijing, and had partners who could be approached. Concerns about 12 maternal health outcomes were identified by literature search and validated using the content validity index. Women and their partners were questioned anonymously about the perceived risks of outcomes after CD and VD before delivery, and the perceived risks of the delivery experienced at 2-3 days after delivery. Perceived risks were compared with reported risks, and factors associated with inaccurate perceptions were evaluated.

**Results:**

Among 272 couples approached, 264 women (97%) and 257 partners (94%) completed the questionnaire both before and after delivery. After CD, the perceived risk of seven health outcomes decreased in women and the perceived risk of two health outcomes increased in partners. After VD, the perceived risk of two outcomes decreased and of one outcome increased in women, and the perceived risk of three outcomes increased in partners. Women perceived higher risks of long-term perineal pain, pelvic organ prolapse, urinary/fecal incontinence, sexual dissatisfaction, and negative impact on the couple’s relationship after VD than after CD (all p < 0.05). CD was the most common factor associated with inaccurate perceptions among women after delivery.

**Conclusions:**

The perceived risks of maternal health outcomes decreased after delivery in women and increased after delivery in their partners. Women continued to have inaccurate perceptions of the risks of health outcomes after delivery, indicating that further education is important.

## Background

The rate of cesarean delivery (CD) has been increasing worldwide, and is now higher than the upper limit of 15% recommended by the World Health Organization (WHO) [1-4]. Although the maternal morbidity associated with CD is not very high, the immediate risks of both minor and severe complications associated with delivery are higher after CD than after vaginal delivery (VD) [4-6]. Long-term consequences associated with CD have been reported, including placental abnormalities, reduced fertility, pelvic floor disorders, sexual dysfunction, and mental distress, but the benefits and risks associated with this mode of delivery are still unclear [5,6]. The decision to perform CD primarily depends on the conditions of the woman and fetus, but can also be affected by the personal preferences of the woman and her partner, the preferences of the woman’s physician, and hospital policy [3,7-9].

Misperceptions regarding maternal health outcomes after CD and VD have led to an increased frequency of CD being performed on demand [10,11]. In a Turkish study, a quarter of 400 women chose CD because of concerns regarding pelvic organ prolapse or stress urinary incontinence after VD [11]. Obviously, previous birth experience may influence a woman’s preference regarding the mode of delivery [7,12,13]. However, few studies have evaluated the perceptions of women regarding the risks of maternal health outcomes after different modes of delivery, and those studies were limited by including women of mixed parity, having small numbers of primiparous women, and not evaluating the perceptions of the women’s partners [13,14].

In China, more than 90% of women deliver at healthcare facilities, and this figure was recorded as 100% in Beijing in 2009 [15]. The WHO Global Survey reported that the rate of CD was as high as 46.2% in Beijing and two other cities [4]. Beijing is therefore a suitable setting to perform a study regarding this important public health issue. The aims of this study were to compare the perceived risks of maternal health outcomes among pregnant women and their partners before and after delivery, to evaluate the accuracy of perceptions, and to evaluate factors related to inaccurate perceptions among women after delivery.

## Methods

### Study design and setting

This cohort study was conducted from July to October 2011 in the Department of Obstetrics and Gynecology at the First Hospital of Tsinghua University (FHTU), which is one of the public referral hospitals for the Chaoyang District of Beijing. This center delivers an average of 160 infants per month and has a CD rate of 45%. All the women receive antenatal care from obstetricians. Women who are judged by the obstetricians to have a low risk of complications are attended at birth by midwives, and women who are judged to have a high risk of complications are attended by obstetricians. The mode of delivery is decided according to the individual circumstances. Maternal requests for CD are not permitted.

### Study sample and sample size

All nulliparous pregnant women at 36-40 weeks gestation who were aged 18-45 years, received antenatal care and planned delivery at FHTU, and had partners who could be approached, were invited to participate in the study during their antenatal registration for electronic fetal monitoring, regardless of whether CD or VD was planned. Women who had previously been admitted to hospital because of medical or psychiatric illness, or who had already started labor, were excluded.

This study was part of a thesis about expectant parents’ perceptions of maternal health outcomes and preferences regarding mode of delivery. The sample size was calculated using the formula for two-group comparisons. As the perceptions of maternal health outcomes according to mode of delivery were unknown, the preference for mode of delivery was used to calculate sample size. In a pilot study of 30 women, 10% preferred CD during pregnancy and 25% preferred CD after delivery. Considering a type I error of 0.05, power of 80%, 5% estimated non-response rate, and 10% estimated loss to follow-up, we calculated that at least 265 couples should be included in the study.

### Development of the questionnaire

The relevant literature was searched to identify maternal health outcomes that might concern expectant parents, including common concerns (such as pelvic organ prolapse and sexual dissatisfaction) and concerns specifically associated with CD or VD. Twelve outcomes were identified, including physical, sexual, and mental health outcomes. A semi-structured questionnaire was developed to evaluate the subjects’ perceived risks of these outcomes. The questionnaire was evaluated by three experts in reproductive health, and was assessed using the content validity index, which ranged from 0.91 to 0.97 among these three experts. This was considered to indicate that the questions were appropriate, as a content validity index of 0.8 or higher is generally considered acceptable [16]. To assess comprehension, the questionnaire was translated into Chinese and was used to question a pilot group of 30 women with similar characteristics. After modification to increase the ease of comprehension, the final version was used for data collection.

### Data collection

The women were interviewed by the principal investigator (WYL), using the questionnaire including three parts of baseline characteristics, Edinburgh Postnatal Depression Scale (EPDS), and perceived risks of maternal health consequences. The women’s partners completed the questionnaire by interview or by themselves at home, depending on whether they were present at the antenatal visit. When the women took the questionnaires home to their partners, they were instructed not to discuss it or give comments while their partners were answering the questions.

The women and their partners were informed that the same questions would be asked twice: during pregnancy and 2-3 days after delivery. The expectant parents were given information about labor and delivery, and the indications for CD and VD. During pregnancy, the expectant parents were questioned regarding their perceived risk of each of the health outcomes after both CD and VD. After delivery, the new parents were questioned regarding their perceived risk of each of the health outcomes after the mode of delivery they had experienced. Completion of the questionnaire took approximately 20 minutes.

### Variables

The primary study outcome was the difference of perceived risks of the 12 maternal health outcomes after CD or VD by the women and their partners during pregnancy and after delivery. Twelve common concerns regarding outcomes after childbirth were considered: wound infection, chronic abdominal pain, long-term perineal pain, pelvic organ prolapse, urinary incontinence, fecal incontinence, abdominal adhesions, dyspareunia, sexual dissatisfaction, negative impact on the couple’s relationship, limitations to daily life/work activities, and negative impact on mental health.

The women and their partners were asked about the perceived risk of each health outcome with the question: “Are you concerned about the possibility of this outcome after delivery, in the short or long term?” If they answered “No”, the perceived risk was recorded as 0%. If they answered “Yes”, they were asked to indicate the perceived risk of the outcome on a scale from 0% to 100%.

Background characteristics were recorded for both the women and their partners. The characteristics recorded for the women included demographic and socioeconomic characteristics (age, level of education, current occupation, monthly family income, whether they were registered as resident in Beijing, family type, and whether they had health insurance for delivery), depression status, whether they had discussed health outcomes with their partner or other people, satisfaction with the couple’s relationship, satisfaction with the antenatal services, satisfaction with the delivery services, satisfaction with the postpartum services, satisfaction with the birth experience, adjustment to being a new mother, and satisfaction with family support. The characteristics recorded for partners were similar, except that they were not asked about depression status, satisfaction with the couple’s relationship, satisfaction with healthcare services, or satisfaction with family support.

The cutoff point of 900 USD was used for family income, which is double the average income per capita in urban Beijing [17]. Depression status was defined according to the validated Chinese version of the Edinburgh Postnatal Depression Scale (EPDS), using a score of 10 or more to indicate depression [18]. Attitudes were rated using the seven-point bipolar Semantic Differential adjectives scales described by Osgood (unsatisfied–satisfied, difficult–easy, or unbearable–bearable).

### Statistical analysis

Data were double-entered and validated by Epidata 3.1, and analyzed using R Software version 2.15.1, 2012 (The R Foundation for Statistical Computing, Vienna, Austria). The seven-point attitude scale scores were dichotomized into ‘negative’ (score 0-4) or ‘positive’ (score 5-7). The perceived risks of outcomes were tested for normal distribution. Perceptions were compared before and after delivery using the paired *t*-test or Wilcoxon signed-rank test, as appropriate. Perceptions were compared between the two modes of delivery using the unpaired *t*-test or Wilcoxon rank sum test, as appropriate.

It was considered clinically and practically meaningful to evaluate whether the parents’ perceptions were accurate. Therefore, the perceived risk of each health outcome among women after delivery was classified into three categories (underestimated, accurately estimated, or overestimated) according to the range of the risk of that outcome reported in the literature. If no range was available, the calculated 95% confidence interval was used instead (Table 1) [19-33]. Both overestimation and underestimation were regarded as inaccurate perceptions. Associations between variables and the accuracy of perceived risk were analyzed using the chi-square test or Fisher’s exact test, as appropriate. Variables with a p value of less than 0.2 were entered into a multinomial regression model using ‘accurately estimated’ as the reference group. Outcomes with a perceived risk of 0% were not analyzed. Possible confounding factors and interactions were checked. A p-value of less than 0.05 was considered significant.

**Table 1:** Reported risks of maternal health outcomes after vaginal and cesarean delivery

**Maternal outcome**	**Vaginal delivery**	**Cesarean delivery**
Wound infection	4.9-6.3% [18]	2-16% [19]
Chronic abdominal pain	0.5-3.8% [20]	2.3-7.3% [20]
Long-term perineal pain	3-15% [21]	0.2-1% [21,22]
Pelvic organ proplapse	1.1-1.2% [23]	0.19-0.25% [23]
Urinary incontinence	18-27% [24]	13-20% [24]
Fecal incontinence	3-13.9% [25,26]	4-5.4% [25,26]
Abdominal adhesions	12-60% [27,28]^a^	24-73% [28]
Dyspareunia	20-56% [26,29,30]	19-34% [26,29]
Sexual dissatisfaction	40-65% [31,32]	35-60% [31,32]
Negative impact on the couple’s relationship	15.6-20.2% [22]	14.9-25.3% [22]
Limitations to daily life/work activities	11.3-19.3% [18]	18.6-32% [18]
Negative impact on mental health	7-30% [30]	7-30% [30]

^a^Calculation based on the adjusted odds ratio of adhesions between cesarean and vaginal delivery being 2.1 (95% CI 1.8–2.4).

### Ethical considerations

The study protocol was approved by the Ethical Committee of the Faculty of Medicine, Prince of Songkla University, Songkhla, Thailand and the Ethical Committee of FHTU, Beijing, China. All participants were provided with information about the study, and gave written informed consent for inclusion before the first interview. Involvement in the study did not place the participants at any risk. Data were collected anonymously to ensure confidentiality.

## Results

A total of 272 couples were approached. The response rates were 100% for the women and 97% for their partners during pregnancy, and 97% for both after delivery. During pregnancy, 3% of the partners could not be approached. After delivery, non-responses were due to couples returning to their homes outside Beijing. The questionnaire was completed both during pregnancy and after delivery by 264 women (97%) and 257 partners (94%). Twenty women planned CD before delivery, and the remainder planned VD.

### Parents’ demographic and socioeconomic characteristics

Table 2 shows the background characteristics of the women. The CD rate was 52.3%. Of all the demographic and socioeconomic characteristics evaluated, the age of the woman was the only factor that was significantly different between women who delivered by CD (median 27 years, interquartile range 24-30 years) and those who delivered by VD (median 26 years, interquartile range 23-29 years) (p = 0.04). The median age of the partners was 28 (IQR, 25-32) years and 138 (53.7%) had a bachelor degree or higher education. Nearly half (43.6%) were local citizens and the majority (94.2%) were employed. None of these partners’ characteristics were significantly different between CD and VD (p > 0.05).

**Table 2:** Baseline characteristics of women who gave birth at FHTU, Beijing, China

**Characteristic**	**n**	**%**
Total	264	100
Age (years)		
<20	6	2.3
20-34	248	93.9
≥35	10	3.8
Education		
≤ Middle school	46	17.4
High school	83	31.4
≥ Bachelor’s degree	135	51.1
Occupation during pregnancy		
Housewife	112	42.4
Private company employee	82	31.1
Government worker	48	18.2
Self-employed	22	8.3
Monthly family income ≥ 900 USD	122	46.2
Registered as resident in Beijing	80	30.3
Nuclear family type	150	56.8
Delivery subsidized by insurance	67	25.4
Discussion of health outcomes		
With partner	112	42.4
With other family members	118	44.7
With friends	148	56.1
With doctor	17	6.4
With nobody	75	28.4
Feeling depressed during pregnancy	96	36.4

### Women’s satisfaction regarding family relationships and healthcare services

Although the majority of women were satisfied with the couple’s relationship (92.8%) and their family support (96.2%), 60.2% reported difficulties in adjusting to being a new mother. The rates of satisfaction with the antenatal, delivery, and postpartum services were 75.8%, 72.0%, and 83.3%, respectively. Only 43 women (16.3%) described their birth experience as bearable. These factors were not significantly different between the CD and VD groups.

### Parents’ perceived risks of maternal health outcomes during pregnancy and after delivery

The perceived risks of maternal health outcomes before and after delivery among women and partners were shown in Table 3 and Table 4 respectively. After delivery, the perceived risks were significantly lower among women and higher among partners than during pregnancy although the concerned maternal health outcomes were different between women and partners. However, among both women and their partners, the median value for the perceived risk of wound infection was 10% during pregnancy and 20% after VD.

**Table 3:** Women’s perceptions of the risk of each maternal health outcome before and after vaginal and cesarean delivery

**Maernal health outcome**	**Cesarean delivery (n = 138)**			**Vaginal delivery (n = 126)**		
	**Median (IQR) %**			**Median (IQR) %**		
	**Before**	**After**	**p-value**^**‡**^	**Before**	**After**	**p-value**^**‡**^
Wound infection	30 (10, 50)	10 (0, 30)	<0.001	10 (0, 20)	20 (10, 30)	0.01
Chronic abdominal pain	30 (10, 50)	20 (0, 40)	0.04	10 (0, 20)	10 (0, 20)	0.55
Long-term perineal pain	0 (0, 10)	0 (0, 0)	0.01	10 (0, 29)	10 (0, 20)	0.65
Pelvic organ prolapse	0 (0, 20)	0 (0, 10)	0.28	10 (0, 30)	10 (0, 20)	0.54
Urinary incontinence	10 (0, 20)	0 (0, 10)	<0.001	10 (0, 30)	0 (0, 18)	0.04
Fecal incontinence	0 (0, 10)	0 (0, 0)	<0.001	0 (0, 20)	0 (0, 10)	0.36
Abdominal adhesions	13 (0, 40)	10 (0, 30)	0.20	0 (0, 10)	0 (0, 10)	0.86
Dyspareunia	0 (0, 20)	5 (0, 20)	0.95	10 (0, 25)	10 (0, 24)	0.65
Sexual dissatisfaction	0 (0, 10)	0 (0, 10)	0.23	20 (0, 40)	10 (0, 20)	0.03
Negative impact on the couple’s relationship	0 (0, 10)	0 (0, 0)	0.01	0 (0, 10)	0 (0, 10)	0.96
Limitations to daily life/work activities	8 (0, 20)	0 (0, 10)	0.06	0 (0, 0)	0 (0, 10)	0.46
Negative impact on mental health	0 (0, 20)	0 (0, 10)	0.007	0 (0, 10)	0 (0, 10)	0.83

^**‡**^p-value for Wilcoxon signed rank test, before versus after delivery.

IQR: interquartile range.

**Table 4:** Partners’ perceptions of the risk of each maternal health outcome before and after vaginal and cesarean delivery

**Maternal health outcome**	**Cesarean delivery (n =135)**			**Vaginal delivery (n =122)**		
	**Median (IQR) (%)**			**Median (IQR) (%)**		
	**Before**	**After**	**p-value**^**‡**^	**Before**	**After**	**p-value**^**‡**^
Wound infection	40 (10,50)	30 (15,50)	0.07	10 (0,20)	20 (10,30)	<0.001
Chronic abdominal pain	20 (10,43)	30 (10,50)	0.12	10 (0,20)	20 (0,30)	0.03
Long-term perineal pain	0 (0,20)	10 (0,20)	0.39	10 (0,30)	10 (0,20)	0.50
Pelvic organ prolapse	10 (0,20)	10 (0,20)	0.25	10 (0,30)	10 (0,20)	0.91
Urinary incontinence	0 (0,20)	10 (0,20)	0.03	10 (0,30)	10 (0,30)	0.21
Fecal incontinence	0 (0,10)	10 (0,20)	0.21	0 (0,20)	10 (0,20)	0.52
Abdominal adhesions	20 (0,30)	20 (8,40)	0.13	0 (0,10)	10 (0,20)	0.06
Dyspareunia	5 (0,20)	10 (0,30)	0.03	10 (0,20)	10 (0,24)	0.91
Sexual dissatisfaction	0 (0,20)	10 (0,20)	0.64	10 (0,20)	10 (0,20)	0.80
Negative impact on the couple’s relationship	0 (0,10)	0 (0,10)	0.50	0 (0,10)	0 (0,10)	0.59
Limitations to daily life/work activities	10 (0,30)	10 (0,30)	0.38	0 (0,10)	10 (0,14)	0.01
Negative impact on mental health	10 (0,30)	10 (0,30)	0.79	0 (0,10)	10 (0,20)	0.05

^**‡**^p-value for Wilcoxon signed rank test, before versus after delivery.

IQR: interquartile range.

### Parents’ perceived risks of maternal health outcomes after CD or VD

**Figure 1:**
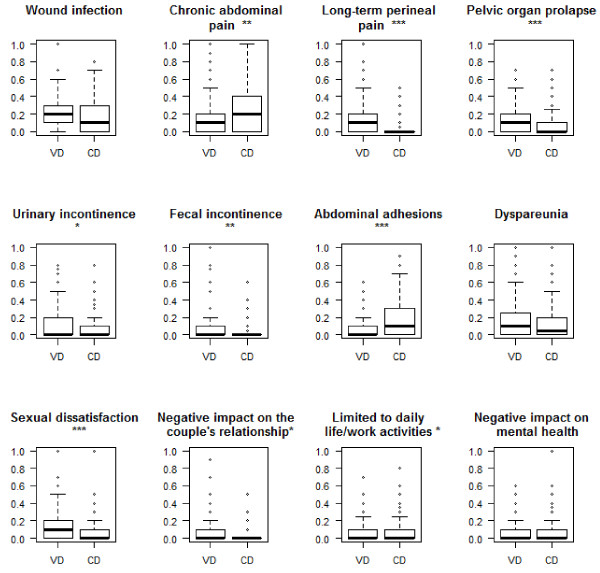
**Women’s perceived risks of maternal health outcomes after delivering by CD or VD.** *p < 0.05, **p < 0.01, ***p < 0.001. VD: vaginal delivery; CD: cesarean delivery.

Figure 1 compared the perceived risks of maternal health outcomes between women delivered by CD and those undergone VD. Among the partners, the perceived risks of wound infection, chronic abdominal pain, abdominal adhesions, limitations to daily life/work activities, and negative impact on mental health were significantly higher after CD than after VD.

### Factors associated with inaccurate perceptions of the risks of health outcomes among women after delivery

Table 5 shows the proportions of women who underestimated, accurately estimated, and overestimated the risk of each of the health outcomes after CD and VD. The proportion of women who accurately perceived the risk of each outcome, compared with the reported risk of the outcome in the literature, ranged from 0% to 35.7%. Women who delivered by VD were more likely to have accurate perceptions than those who delivered by CD.

**Table 5:** **Accuracy of women’s perceived risks of health outcomes after cesarean and vaginal delivery, compared with the reported risks in Table**1

**Perception of outcome**	**n (%)**		
	**VD**	**CD**	**p-value**
	**n = 126**	**n = 138**	
Wound infection			<0.001
Underestimated	26 (20.6)	47 (34.1)	
Accurate	1 (0.8)	24 (17.4)	
Overestimated	99 (78.6)	67 (48.6)	
Chronic abdominal pain			0.11^‡^
Underestimated	52 (41.3)	43 (31.2)	
Accurate	0 (0)	1 (0.7)	
Overestimated	74 (58.7)	94 (68.1)	
Long-term perineal pain			<0.001
Underestimated	42 (33.3)	105 (76.1)	
Accurate	40 (31.7)	0 (0)	
Overestimated	44 (34.9)	33 (23.9)	
Pelvic organ prolapse			<0.001
Underestimated	50 (39.7)	89 (64.5)	
Accurate	0 (0)	0 (0)	
Overestimated	76 (60.3)	49 (35.5)	
Urinary incontinence			0.44
Underestimated	94 (74.6)	112 (81.2)	
Accurate	12 (9.5)	10 (7.2)	
Overestimated	20 (15.9)	16 (11.6)	
Fecal incontinence			<0.001
Underestimated	80 (63.5)	108 (78.3)	
Accurate	21 (16.7)	2 (1.4)	
Overestimated	25 (19.8)	28 (20.3)	
Abdominal adhesions			0.06^‡^
Underestimated	101 (80.2)	96 (69.6)	
Accurate	25 (19.8)	40 (29.0)	
Overestimated	0 (0)	2 (1.4)	
Dyspareunia			0.002
Underestimated	74 (58.7)	93 (67.4)	
Accurate	45 (35.7)	26 (18.8)	
Overestimated	7 (5.6)	19 (13.8)	
Sexual dissatisfaction			0.30^‡^
Underestimated	110 (87.3)	128 (92.8)	
Accurate	13 (10.3)	9 (6.5)	
Overestimated	3 (2.4)	1 (0.7)	
Negative impact on the couple’s relationship			0.007
Underestimated	102 (81.0)	126 (91.3)	
Accurate	9 (7.1)	9 (6.5)	
Overestimated	15 (11.9)	3 (2.2)	
Limitations to daily life/work activities			0.09
Underestimated	91 (72.2)	107 (77.5)	
Accurate	13 (10.3)	19 (13.8)	
Overestimated	22 (17.5)	12 (8.7)	
Negative impact on mental health			0.58
Underestimated	83 (65.9)	93 (67.4)	
Accurate	37 (29.4)	35 (25.4)	
Overestimated	6 (4.8)	10 (7.2)	

^**‡**^p-value for Fisher’s exact test, others for chi-square test.

The factors associated with the accuracy of the perceived risk of each health outcome by multinomial regression analysis are shown in Table 6. The mode of delivery was the factor most commonly associated with inaccurate perceptions of the risks of health outcomes. Both over-and under-estimation of the health outcomes were lower for wound infection and higher for fecal incontinence and dyspareunia among women who delivered by CD than those who delivered by VD. Satisfaction with the delivery services, discussion of health outcomes with their partner or other family members, monthly family income, and residency status in Beijing were also associated with inaccurate perceptions of the risks of health outcomes. There was an interaction between high monthly family income and being satisfied with the delivery services, and the perceived risk of negative impact on mental health. Each of these factors was associated with a high risk of underestimation of the negative impact on mental health, but when considered together, the risk was lower (relative risk = 0.16, p < 0.01).

**Table 6:** Factors associated with women’s inaccurate perceptions of the risks of health outcomes after delivery

**Perception of outcome**	**Associated factor**	**Underestimated**	**Overestimated**
		**RRR (95% CI)**	**ref. = accurate**
**Wound infection**^**#1**^			
	Cesarean delivery	0.09 (0.01-0.67)^*^	0.03 (0-0.21)^‡^
**Fecal incontinence**			
	Not on the Beijing registry	3.11 (1.23-7.9)^*^	1.77 (0.64-4.95)
	Cesarean delivery	15.54 (3.50-69.01)^‡^	12.36 (2.61-58.42)^†^
**Dyspareunia**			
	Satisfied with delivery services	1.23 (0.65-2.35)	0.33 (0.13-0.85)^*^
	Cesarean delivery	1.95 (1.09-3.49)^*^	5.03 (1.82-13.9)^†^
**Sexual dissatisfaction**			
	Discussion with partner	0.25 (0.07-0.87)^*^	0.67 (0.03-13.02)
**Negative impact on the couple’s relationship**			
	Cesarean delivery	1.24 (0.47-3.23)	0.20 (0.04-0.94)^*^
**Limitations to daily life/work activities**			
	Discussion with other family member	0.41 (0.19-0.88)^*^	0.68 (0.25-1.8)
**Negative impact on mental health**			
	High monthly family income	3.17 (1.05-9.51)^*^	8.75 (1.34-57)^*^
	Satisfied with delivery services	4.33 (1.96-9.57)^‡^	1.17 (0.15-9.14)
	High income and satisfied with services^#2^	0.16 (0.04-0.57)^‡^	0.27 (0.02-3.31)

RRR: relative risk ratio; ref.: reference.

^*^p < 0.05, ^†^p < 0.01, ^‡^p < 0.001.

^#1^Results adjusted for level of education.

^#2^There was interaction between these two variables.

## Discussion

Limited information and lack of childbirth experience resulted in misperceptions regarding maternal health outcomes after CD and VD by both women and their partners. Women who delivered by VD were more concerned about perineal pain, pelvic floor disorders, sexual dissatisfaction, and negative impact on the couple’s relationship than those who delivered by CD. The perceived risks of these outcomes remained inaccurately high after delivery.

There were significant changes in the perceived risks among women after delivery. No previous studies have evaluated differences in these perceptions before and after childbirth according to the mode of delivery. Only two previous cross-sectional studies evaluated the perceived risks and benefits of modes of delivery among pregnant women, and they concluded that the women knew more about the benefits of CD than the risks [13,14]. However, one of these studies included only 20 women who preferred CD [13]. The other study evaluated the knowledge of women about CD and VD, but the lists provided to women overemphasized the safety of CD and the risks of VD [14].

In this study, the significant changes in the perceived risks of health outcomes among women all indicated reduced concern after delivery, except for the perceived risk of wound infection after VD. Fear of childbirth during pregnancy may lead to unknown anxiety [34] and an over-estimation of the risks, as shown in this study the concerns were reduced immediately after delivery. In contrast, the increase in the perceived risk of wound infection after VD may have been caused by fear of contamination of the perineal wound after routine episiotomy. The differences in the perceived risks of health outcomes between women and their partners might be attributable to marginalized involvement of the partners [35].

In this study, inaccurate perceptions among women were related to the mode of delivery, lack of satisfaction with delivery services, and socioeconomic factors. Accurate perception of the risk of most health outcomes was lower among women who delivered by CD than those who delivered by VD. The overestimation of negative outcomes after VD and underestimation of negative outcomes after CD suggest that women overestimate the risks of VD compared with CD. This is consistent with the findings of a Cochrane review that the information offered to pregnant women about CD might be insufficient [36].

Lack of satisfaction with the delivery services and lack of discussion with their partner or family members may reflect inadequate obstetric care and health system responsiveness [37,38]. Although most of the women in this study were satisfied with the healthcare services, the majority of them considered their birth experience to have been unbearable, which is a sign of inadequate obstetric care [37]. This inconsistency between the high rates of satisfaction and the low rate of tolerable birth experience may reflect a reluctance among women to criticize their obstetric care [39]. As expectant mothers desire support from their partners during pregnancy and childbirth, it is also important to educate expectant fathers to ensure that they can provide this support [40].

Socioeconomic factors such as residency status in Beijing and the monthly family income were associated with inaccurate perceptions among women, which may reflect inequities in access to obstetric care [3]. Similar to other urban settings in China [4], the high CD rate in this study is of concern to many researchers, and has been reported to be associated with the supply of health services and economic factors [3,41].

This is the first cohort study that assessed the perceived risks of maternal health outcomes among both pregnant women and their partners, and compared perceptions during pregnancy and after delivery as well as between CD and VD. The accuracy of the perceived risk of each health outcome compared with its reported risk was also assessed. However, this study has some limitations. First, only 12 maternal health outcomes were assessed. However, these 12 outcomes were either common concerns (such as pelvic organ prolapse and sexual dissatisfaction) or concerns that are associated with the mode of delivery (such as chronic abdominal pain), and included physical, sexual, and mental health outcomes. Second, the generalizability of the findings may be limited because this study was conducted in a referral hospital with a relatively high proportion of patients with medium to high socioeconomic status. However, our analysis considered the interquartile range for the perceived risk of each health outcome, and therefore covers a range of perceptions. Third, the studies that were used to obtain the previously reported risks of health outcomes may have had varying methodologies. Some studies had small sample sizes and did not report confidence intervals, and we sometimes had to calculate the confidence interval using the results of only one study. For abdominal adhesions after vaginal delivery, the reported range of risk was based on the odds ratio of adhesions between women with cesarean and vaginal deliveries. Finally, we did not ask the participants why their perceptions were different before and after delivery or between CD and VD. We could therefore not determine whether these changes resulted from their own judgments and birth experience, or the opinions of their caregivers, or both. Further studies are needed to definitively determine the risks of the relevant maternal health outcomes after CD and VD. In addition, further interventional studies are needed to improve the perceptions regarding the risks of these health outcomes among both pregnant women and their partners.

## Conclusions

The perceived risks of maternal health outcomes decreased after delivery in women and increased after delivery in their partners. Among women, perceptions regarding the risks of maternal health outcomes continued to be inaccurate even after delivery, and this inaccuracy was associated with the mode of delivery. More information about birth procedures and health outcomes should be provided to both women and their partners.

## Competing interests

The authors declare that they have no competing interests.

## Authors’ contributions

WYL and TL contributed to study design, data analysis and interpretation, and writing of the manuscript. WYL organized and coordinated the data collection. BSP contributed to study design, data interpretation, and writing of the manuscript. All authors read and approved the final manuscript.

## Pre-publication history

The pre-publication history for this paper can be accessed here:

http://www.biomedcentral.com/1471-2393/14/12/prepub
